# A Modified Two-Step Screening Strategy for Gestational Diabetes Mellitus Based on the 2013 WHO Criteria by Combining the Glucose Challenge Test and Clinical Risk Factors

**DOI:** 10.3390/jcm7100351

**Published:** 2018-10-13

**Authors:** Katrien Benhalima, Paul Van Crombrugge, Carolien Moyson, Johan Verhaeghe, Sofie Vandeginste, Hilde Verlaenen, Chris Vercammen, Toon Maes, Els Dufraimont, Christophe De Block, Yves Jacquemyn, Farah Mekahli, Katrien De Clippel, Annick Van Den Bruel, Anne Loccufier, Annouschka Laenen, Caro Minschart, Roland Devlieger, Chantal Mathieu

**Affiliations:** 1 Department of Endocrinology, University hospital Gasthuisberg, KU Leuven, 3000 Leuven, Belgium; carolien.moyson@uzleuven.be (C.Mo.); caro.minschart@kuleuven.be; (C.Mi.); chantal.mathieu@uzleuven.be (C.Ma.); 2 Department of Endocrinology, OLV ziekenhuis Aalst-Asse-Ninove, 9300 Aalst, Belgium; Paul.Van.Crombrugge@olvz-aalst.be; 3 Department of Obstetrics & Gynecology, University Hospital Gasthuisberg, KU Leuven, 3000 Leuven, Belgium; johan.verhaeghe@uzleuven.be (J.V.); roland.devlieger@uzleuven.be (R.D.); 4 Department of Obstetrics & Gynecology, OLV Ziekenhuis Aalst-Asse-Ninove, 9300 Aalst, Belgium; Sofie.Vandeginste@olvz-aalst.be (S.V.); Hilde.Verlaenen@olvz-aalst.be (H.V.); 5 Department of Endocrinology, Imelda ziekenhuis, 2820 Bonheiden, Belgium; Chris.Vercammen@imelda.be (C.V.); Toon.Maes@imelda.be (T.M.); 6 Department of Obstetrics & Gynecology, Imelda Ziekenhuis, 2820 Bonheiden, Belgium; Els.Dufraimont@imelda.be; 7 Department of Endocrinology-Diabetology-Metabolism, Antwerp University Hospital, 2560 Edegem, Belgium; Christophe.DeBlock@uza.be; 8 Department of Obstetrics & Gynecology, Antwerp University Hospital, 2560 Edegem, Belgium; Yves.Jacquemyn@uza.be; 9 Department of Endocrinology, Kliniek St-Jan Brussel, 1000 Brussel, Belgium; fmekahli@clstjean.be; 10 Department of Obstetrics & Gynecology, Kliniek St-Jan Brussel, 1000 Brussel, Belgium; kdeclippel@gmail.com; 11 Department of Endocrinology, AZ St Jan Brugge, 8000 Brugge, Belgium; Annick.VandenBruel@azsintjan.be; 12 Department of Obstetrics & Gynecology, AZ St Jan Brugge, 8000 Brugge, Belgium; anne.loccufier@azsintjan.be; 13 Center of Biostatics and Statistical bioinformatics, KU Leuven, 3000 Leuven, Belgium; annouschka.laenen@kuleuven.be

**Keywords:** gestational diabetes mellitus, 2013 WHO criteria, risk factors, screening

## Abstract

This study determines if a modified two-step screening strategy with a glucose challenge test (GCT) ≥ 7.2 mmol/L and clinical risk factors improves the diagnostic accuracy for gestational diabetes mellitus (GDM), based on 2013 WHO criteria, while limiting the number of oral glucose tolerance tests (OGTT). This was a prospective multicentric cohort study with 1811 participants receiving both GCT and 75 g OGTT in pregnancy. Participants and health care providers were blinded for GCT. Characteristics were analyzed across four glucose tolerance groups: abnormal (≥7.2 mmol/L), GCT GDM (*n* = 165), normal GCT GDM (*n* = 63), abnormal GCT normal glucose tolerant (NGT) (*n* = 472); normal GCT NGT (*n* = 1113). Compared to normal GCT NGT women, normal GCT GDM women had increased rates of obesity (23.8% vs. 10.5%, *p* < 0.001), ethnic minority background (19.3% vs. 8.2%, *p* < 0.001) and a history of GDM (13.8% vs. 4.6%, *p* = 0.03). By combined screening of GCT ≥ 7.2 mmol/L with these risk factors, sensitivity increased to respectively, 74.1–78.1% using one risk factor, and to 82.9% using any of these risk factors with a specificity of 57.5%. By using a modified two-step screening strategy, the number of women needing both a GCT and OGTT would be reduced to 25.5%, and 52.6% of all OGTTs could be avoided, compared to a universal one-step approach.

## 1. Introduction

Gestational diabetes mellitus (GDM) is an important modifiable risk factor for adverse pregnancy outcomes and is associated with an increased risk of developing type 2 diabetes mellitus in later life [1,2,3]. The ‘International Association of Diabetes and Pregnancy Study Groups’ (IADPSG) recommends a universal one-step approach with the 75 g oral glucose tolerance test (OGTT) for the screening of GDM [4]. Since the adoption of the IADPSG recommendation by the World Health Organization (WHO), the IADPSG criteria are commonly referred to as the 2013 WHO criteria [5]. The IADPSG recommendation remains controversial due to the important increase in GDM prevalence, the increased workload, the need for a fasting test, and the risk for increased medicalization of care [6,7,8]. Moreover, randomized controlled trials (RCTs) showing treatment benefits for GDM used a two-step approach with a 50 g glucose challenge test (GCT) or risk factors [1,2]. Several professional associations therefore still adhere to a two-step approach, using a non-fasting GCT to determine whether an OGTT should be performed [6,9,10]. The GCT is easier to perform, and it is generally better tolerated than an OGTT [11]. In addition, a two-step screening strategy with a GCT could limit the number of OGTTs. The GCT has been used in combination with the 100 g OGTT or the 75 g OGTT with various diagnostic criteria, but data were lacking on the sensitivity and specificity of the GCT in conjunction with the IADPSG/2013 WHO criteria for GDM [12]. We have recently shown that the threshold of the GCT would need to be reduced to at least 7.2 mmol/L, to achieve sensitivity ≥ 70% for GDM, based on the 2013 WHO criteria [13]. By applying a GCT threshold of 7.2 mmol/L, 65.1% of all OGTTs could be avoided, compared to the one-step approach with the 75 g OGTT, but 27.6% of women with GDM would be missed [13]. Our aim was therefore to evaluate the characteristics of women with GDM, who would be missed using a GCT threshold ≥ 7.2 mmol/L, and to determine whether a modified two-step screening strategy with the GCT ≥ 7.2 mmol/L and clinical risk factors could improve the diagnostic strategy while exposing as few women as possible to the burden of an OGTT. We also aimed to evaluate the tolerance of the tests, and which screening strategy women preferred.

## 2. Subjects and Methods

The study was registered in ClinicalTrials.gov (NCT02036619). The study protocol was approved by the Institutional Review Boards of all participating centers (B322201420693). Participants provided informed consent before inclusion in the study. 

### 2.1. Study Design

The ‘Belgian Diabetes in Pregnancy’ study (BEDIP-N) was a prospective, multicenter cohort study. The protocol of the BEDIP-N study was previously published [14]. Women were universally screened by a fasting plasma glucose (FPG) between 6–14 weeks for diabetes (FPG ≥ 7.0 mmol/L) and prediabetes (FPG ≥ 5.5 mmol/L and ≤ 6.9 mmol/L) as defined by the American Diabetes Association (ADA) [9]. Women without diabetes and prediabetes in early pregnancy, were universally screened for GDM between 24–28 weeks of pregnancy, and they received both a 50 g GCT and 75 g OGTT. Participants and health care providers were blinded for the result of the GCT. The GCT was analyzed centrally at the lab of the university hospital of Leuven (UZ Leuven), and only the coordinator of the database had access to the results of the GCT during the study. Because the GCT was not yet validated with the 2013 WHO criteria, and since the result of the GCT was not used to treat patients, GCT thresholds were not pre-specified. The reference standard for the diagnosis of GDM was the 75 g OGTT with the 2013 WHO criteria (FPG ≥ 5.1 mmol/L, 1-h glycaemia ≥ 10.0 mmol/L, 2-h glycaemia ≥ 8.5 mmol/L, diagnosis of GDM if ≥ 1 values is abnormal)] [4]. The analyses of the OGTT were done locally at each center, and the results were available to participants and health care providers. Women with GDM were treated according to a standardized protocol in line with routine clinical practice [14]. The ADA-recommended glycemic targets were used: FPG < 5.3 mmol/L, 1-h after the meal < 7.8 mmol/L or 2-h after the meal < 6.7 mmol/L [9]. If targets were not achieved within two weeks after the start of lifestyle measures, insulin was started [14]. 

### 2.2. Study Participants

The cohort was recruited from seven Belgium centers, two university hospitals, and five non-university centers. Two centers had 2300 deliveries per year, and five centers had between 700–1400 deliveries per year (total of 9700 deliveries per year). Over a three year period, between April 2014 and March 2017, women between 18–45 years who presented for prenatal care between 6–14 weeks of pregnancy, were invited to participate in the study. The most important exclusion criteria were multiple pregnancy, diabetes, and a history of bariatric surgery [14]. 

### 2.3. Study Assessments

At first visit, baseline characteristics (age, pre-pregnancy weight, ethnic background, history of smoking, family history of diabetes (a first- or second-degree relative with diabetes, or a mother or sister with GDM), parity, history of polycystic ovarian syndrome (PCOS), GDM or impaired glucose intolerance) and the obstetrical history were collected [14]. Hypertension was defined as a systolic blood pressure ≥ 140 mmHg and/or diastolic blood pressure ≥ 90 mmHg, overweight as a body mass index (BMI) ≥ 25–29.9 kg/m², and obesity as a BMI ≥ 30 kg/m². At first visit and at the time of the OGTT, anthropometric measurements were obtained, and several self-administered questionnaires were completed [14]. At the time of the GCT and OGTT, a questionnaire on tolerance and preference for the test was completed [14]. 

For the fasting blood sample at first visit, participants had to be fasting for at least 8 h. The GCT was performed between 24–26 weeks, and no specific preparation was necessary. First, a blood sample was collected to evaluate the non-fasting glycaemia, followed by the consumption of a 50 g glucose beverage with the measurement of the plasma glucose level after 1 h. Data on the time of the GCT and the time of the last meal were also collected. The 75 g OGTT was performed between 26–28 weeks. Participants had to be fasting for at least 10 h. The fasting blood collection was followed by blood collections at 30 min, 1 h, and 2 h for the measurement of glucose and insulin. The analyses of the FPG at 6–14 weeks and the glucose measurements of the OGTT were performed locally at each center. The analyses of the GCTs, insulin, lipids, and HbA1c levels were performed centrally at the lab of UZ Leuven, and these results were not communicated to participants and health care providers during the study. 

Different indices of insulin sensitivity (the Matsuda index and the homeostasis model assessment of insulin resistance (HOMA-IR)) and beta-cell function (HOMA-B, the insulinogenic index divided by HOMA-IR and the insulin secretion-sensitivity index-2 (ISSI-2)), were measured, as previously described [14,15,16,17,18,19]. The units of the glucose and insulin measurements used to calculate these indices were respectively mg/dL and pmol/L.

Plasma glucose was measured by an automated colorimetric-enzymatic method on a Hitachi/Roche-Modular P analyzer (Basel, Switzerland). Insulin was measured by the immunometric ECLIA (Roche Modular E170). HbA1c was measured by a Tosoh Automated Glycohemoglobin Analyzer HLC-723G8 (Tosoh Europe, Tessenderlo, Belgium). Lipid levels were measured by the immunoassay analyzer Cobas 8000 modular analyzer series (Roche, Basel, Switzerland). Coefficients of variance were 1% for glucose, 6% for insulin, 2% for lipids, and 2% for HbA1c in the Lab of UZ Leuven.

### 2.4. Statistical Analysis

We calculated the number of women with GDM that would be missed when using a GCT of 7.2 mmol/L, compared to the universal one-step approach with the 75 g OGTT. To evaluate differences in characteristics between different groups based on the GCT ≥ 7.2 mmol/L and 75 g OGTT, participants were stratified into the following four gestational glucose tolerance groups: GDM with an abnormal preceding GCT (abnormal GCT GDM); GDM with a normal preceding GCT (normal GCT GDM); normal glucose tolerance (NGT) on the antepartum OGTT with an abnormal preceding GCT (abnormal GCT NGT); normal glucose tolerance on the OGTT with a normal preceding GCT (normal GCT NGT). We constructed 2 × 2 tables and calculated sensitivity, specificity, positive and negative likelihood ratios (LRs) with 95% confidence intervals (CI), and positive and negative posttest probability rates of screening with a GCT threshold of 7.2 mmol/L combined with clinical risk factors. The clinical risk factors that were combined with the GCT to screen for GDM were selected based on the differences in risk factors between the normal GCT GDM group and the normal GCT NGT group. We analyzed a modified two-step screening approach, which implies that women with a risk factor would receive an OGTT directly without the need of a GCT, while women without a risk factor would undergo a universal two-step approach with the GCT, and only receive an OGTT if the GCT ≥ 7.2 mmol/L. For the sensitivity and specificity analyses of the GCT combined with risk factors, only women who had received both the GCT and OGTT were included in the analyses. 

The influence of season, time since last meal, random glucose value before the GCT, and time of the day the GCT was performed, on the discriminative performance of the GCT, were analyzed by means of logistic regression, testing for the interaction between GCT level and the respective factor.

Continuous variables were presented as mean if normally distributed, as median otherwise, categorical variables as percentage. The Chi-square test was used for comparing groups on categorical variables, the Fisher exact test was used in cases of small cell frequencies (<5). The Mann–Whitney U test or Kruskal–Wallis test was used for comparing two or multiple groups, respectively, on continuous variables. A *p*-value < 0.05 (two-tailed) was considered significant. Analyses were performed by A. Laenen using Analytics Software & Solutions (SAS) software (Cary, NC, USA, version 9.4).

## 3. Results

### 3.1. Study Participants

We recruited 2006 women (8.1% of the total pregnant population at the different centers during the study). Table 1 gives an overview of the baseline characteristics. Of all participants, 10.7% (213) had an ethnic minority (EM) background, of which the most common were Northern-African (33.3%), Asian (18.8%), Turkish (12.2%), Black-African (8.3%), Latin-American (6.6%), and Middle-Eastern (3.7%). Compared to the background Flemish pregnant population, the BEDIP cohort was older, was less often from an EM background, had more often a higher education, and rates of overweight and obesity were slightly higher (Table 2).

**Figure 1:**
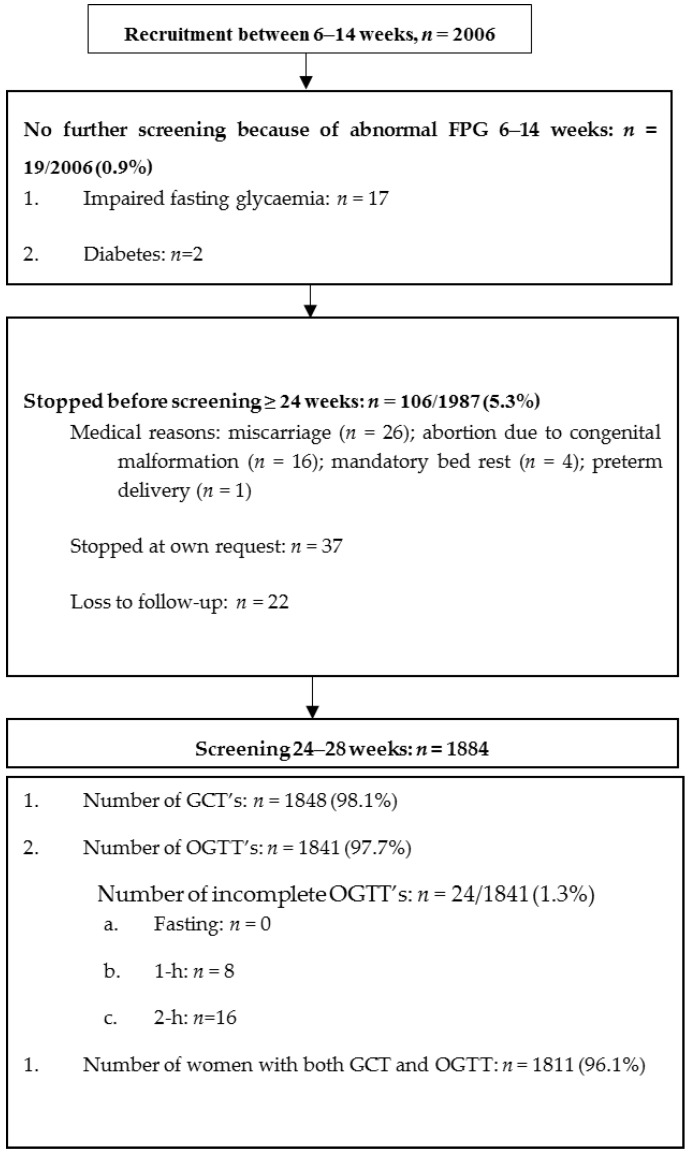
Flow of participants. FPG: fasting plasma glucose; GCT: 50 g glucose challenge test; OGTT: 75 g 2 -h oral glucose tolerance test.

Of the total cohort, 19 (0.9%) participants were excluded from further screening because of diabetes (2) or prediabetes (17) at first visit, and 106 (5.3%) participants stopped before 24 weeks of pregnancy because of a medical reason (47), stopped at own request (37), or were loss to follow-up (22) (Figure 1). Of the 1884 participants receiving further screening at 24–28 weeks of pregnancy, 96.1% (1811) received both a GCT and OGTT. The GCT was performed at a mean of 24.5 weeks and the 75 g OGTT at a mean of 26.9 weeks (Table 1). 

Based on the universal one-step approach with the 75 g OGTT and the 2013 WHO criteria, GDM prevalence was 12.5% (231). Of the 231 women with GDM, 75.3% (174) had one abnormal value, 20.8% (48) had two abnormal values, and 3.9% (9) had three abnormal values. Of the 174 women with GDM based on one abnormal value, 28.7% (50) had an abnormal FPG, 23.6% (41) had an abnormal 1-h value, and 47.7% (83) had an abnormal 2-h value. The GDM group was older, had a higher BMI, had more often an EM background, had more often a family history with diabetes and a history of GDM, and had higher levels of FPG, HbA1c, triglycerides, and HOMA-IR at first visit (Table 1). 

### 3.2. Characteristics of Women with GDM Who Would be Missed Using a GCT Threshold of 7.2 mmol/L

**Figure 2:**
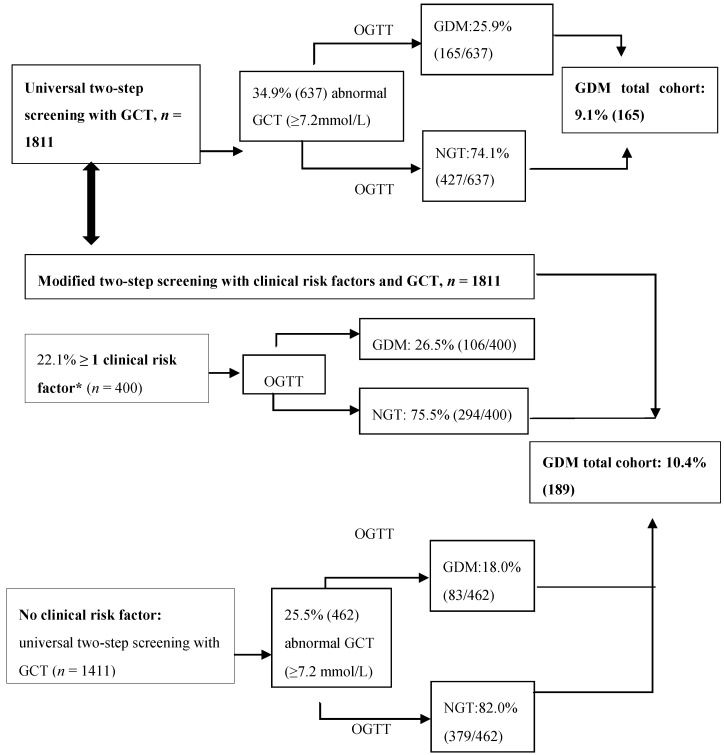
Universal two-step screening strategy with GCT ≥ 7.2 mmol/L, compared to the modified two-step screening strategy with GCT and clinical risk factors. GCT: 50 g glucose challenge test; OGTT: 75 g 2-h oral glucose tolerance test; GDM: gestational diabetes mellitus; NGT: normal glucose tolerance; * clinical risk factors: BMI ≥ 30 kg/m²; an ethnic minority background or a history of gestational diabetes mellitus.

Of all women with GDM (231), 228 women received both the GCT and 75 g OGTT. By using a two-step strategy with the GCT threshold of 7.2 mmol/L, the prevalence of GDM would be 9.1% (165), and 27.6% (63) of all women with GDM would be missed compared to the one-step approach with the 75 g OGTT (Figure 2). Compared to the normal GCT NGT group, the abnormal GCT GDM group had many clinical risk factors (Table 3). In contrast, the normal GCT GDM group had only significantly increased rates of obesity (23.8% vs. 10.5%, *p* < 0.001), an EM background (19.3% vs. 8.2%, *p* < 0.001) and a history of GDM (13.8% vs. 4.6%, *p* = 0.03) compared to the normal GCT NGT group (Table 3).

### 3.3. Sensitivity of Screening with the GCT Combined with Clinical Risk Factors

If screening with a GCT using a 7.2 mmol/L threshold would be combined with the clinical risk factors obesity, EM background and a history of GDM, sensitivity would improve to 74.1% (95% CI 67.9–79.7%)−78.1% (95% CI 72.1–83.3% ) when one risk factor was used, and sensitivity would further increase to 82.9% (95% CI 77.4–87.5%) using any of these three risk factors, with a specificity of 57.5% (95% CI 55.0–59.9%). In addition, the number of women with GDM that would be missed would be reduced to 17.1% (39); the number of women needing both a GCT and OGTT would be reduced to 25.5%, and 52.6% of all OGTT’s could still be avoided (Table 4 and Figure 2). 

The diagnostic accuracy of the GCT was not influenced by season (*p* = 0.54) nor by the time after the last meal before the GCT (*p* = 0.26) or by the random glucose value before the GCT (*p* = 0.73). The global interaction with time of testing during the day of the GCT was not significant (*p* = 0.06) but the GCT was more often positive (≥7.2 mmol/L) in the afternoon (>12:00 a.m., 40.6%) compared to the morning (<12:00 a.m., 28.1%, *p* < 0.001). The positive post-test probability of the GCT was highest when the GCT was performed <12:00 a.m. (33.4%) with an AUC for the GCT of 0.82 (0.77–0.86) compared to when the GCT was performed >12:00 a.m. (22.9%) with an AUC of 0.74 (0.69–0.79). 

Analyses of a GCT threshold at which GDM could be diagnosed without proceeding to the OGTT, showed a specificity of 99.9% at a threshold of 11.1 mmol/L, a sensitivity of 3.1%, with eight women (0.4%) ≥ this threshold. The lowest GCT threshold with a specificity of at least 99.1%, was 10.2 mmol/L with a sensitivity of 7.9% and 33 women (1.8%) ≥ this threshold. 

### 3.4. Differences in Biochemical Variables Across the Four Subgroups According to the GCT and OGTT Result

The FPG and triglyceride levels at first visit and the 1 h and 2 h glucose levels on the OGTT, and the triglyceride levels at the time of the OGTT, were significantly higher in both GDM groups compared to the normal GCT GDM group (Table 3). The 1 and 2 h glucose values on the OGTT were significantly higher in the abnormal GCT GDM group compared to the normal GCT GDM group, but the indices of insulin sensitivity and beta-cell function were not significantly different between both GDM groups, and this was significantly lower compared to the normal GCT NGT group (Table 3). 

Maternal age, cholesterol, and triglyceride levels at first visit were significantly higher while FPG and HOMA-IR at first visit were not significantly different between the abnormal GCT NGT and normal GCT NGT groups. The 1 -h and 2 -h glucose values on the OGTT were higher, and the indices of insulin sensitivity and beta-cell function based on the OGTT were significantly lower in the abnormal GCT NGT group, compared to the normal GCT NGT group (Table 3). 

### 3.5. Tolerance and Preference of the GCT and OGTT

Evaluation of the tolerance of the tests showed that 20.6% (377) of women had one or more complaints about the GCT, including nausea in 45.1% (170), dizziness or a feeling of fainting in 38.2% (144), a bad taste in 27.3% (103), abdominal pain in 2.6% (10), and vomiting in 2.4% (9) women. Of all women, 43.4% (784) had one or more complaints about the OGTT, including nausea in 55.5% (434), dizziness or a feeling of fainting in 48.5% (380), a bad taste in 29.6% (232), abdominal pain in 5.0% (39), and vomiting in 4.5% (35). 

Of all participants receiving screening ≥ 24 weeks of pregnancy, 41.6% (750) indicated that they felt that it was difficult to be fasting. An evaluation of which screening test and screening strategy was preferred, showed that 54.9% (987) preferred the GCT, 6.2% (112) preferred the OGTT, 38.9% (700) had no preference, while 46.3% (833) indicated that they preferred a two-step screening strategy with only a OGTT when the GCT was abnormal, 26.2% (471) preferred a one-step diagnostic approach with the 75 g OGTT, and 27.5% (494) had no preference. 

## 4. Conclusions

We show now that the GDM group that would be missed when using a universal two-step screening strategy with a GCT threshold of 7.2 mmol/L and diagnosis of GDM based on the 2013 WHO criteria, was more often obese, more often had an EM background and a history of GDM compared to the normal GCT NGT group. A modified two-step screening strategy with the GCT and clinical risk factors increased the sensitivity to 82.9%, and 52.6% of all OGTTs could be avoided, compared to the one-step approach. A modified two-step screening strategy might therefore be a practical alternative to the universal one-step approach with 75 g OGTT. 

## 5. Discussion

We demonstrate that women with GDM that would be missed by using a universal two-step screening strategy with the GCT threshold of 7.2 mmol/L, and diagnosis of GDM based on the 2013 WHO criteria, were more often obese, and more often had an EM background and a history of GDM compared to the normal GCT NGT group. A modified two-step screening strategy with GCT ≥ 7.2 mmol/L and these clinical risk factors increased sensitivity to 82.9% and reduced the number of women with GDM that would be missed to 17.1%. In addition, the number of women needing both a GCT and OGTT would be reduced to 25.5%, and 52.6% of all OGTTs could still be avoided, compared to the one-step approach. We found no significant interactions of season, time of testing during the day, time since the last meal, or the random glucose value before the GCT, on the diagnostic accuracy of the GCT. Moreover, the GCT was better tolerated than the OGTT, and more women preferred a two-step strategy. 

We have recently shown that applying lower thresholds to the GCT than 7.2 mmol/L for a subsequent OGTT, would increase sensitivity rates to ≥ 77%, but 40–50% of pregnant women would need a subsequent OGTT at a GCT threshold between 6.9 mmol/L to 6.7 mmol/L [13]. Combined screening by the GCT ≥ 7.2 mmol/L and clinical risk factors, could therefore improve the identification of women who would need a OGTT without first the need of a GCT based on their risk profile, and as such, this would limit the number of women who would need both a GCT and OGTT, while at the same time limit the total number of OGTTs needed. The sensitivity of the proposed modified two-step screening strategy with the 2013 WHO criteria is in line with the generally reported sensitivity rates of the GCT with the 100 g OGTT, and the Carpenter and Coustan criteria, with a risk of missing the diagnosis <10–20%, which is generally considered to be acceptable for a screening strategy [12]. A modified two-step screening strategy with a GCT and clinical risk factors might therefore be a practical alternative to the universal one-step approach with the 75 g OGTT, to reduce the workload and the need for a fasting test in about 50% of women. 

The GCT has been used in combination with the 100 g OGTT or the 75 g OGTT with various diagnostic criteria such as the Carpenter & Coustan criteria, the National Diabetes Data Group (NDDG) criteria, the 1999 WHO criteria or the Canadian Diabetes Association criteria, and has shown variable sensitivity rates between 70–88% and specificity rates between 69–89% when using a GCT threshold of 7.8 mmol/L and sensitivity rates between 88–99% and specificity rates between 66–77% when using a GCT threshold of 7.2 mmol/L [12]. Another systematic review showed that for screening based on risk factors, the sensitivity of the GCT was 74% for a specificity of 77%, while for universal screening, the sensitivity of the GCT was 74% for a specificity of 85% [21]. However, up to 80% of the studies in these systematic reviews had a high or unclear bias, because the result of the screening test was used to determine whether further testing was needed for GDM and not all patients received a confirmatory OGTT if the GCT was below a certain threshold [12,21]. Our study avoided these limitations since both healthcare providers and participants were blinded for the GCT, and all participants received an OGTT irrespective of the GCT result. 

Moreover, several variables that may potentially influence the accuracy of the GCT were analyzed and we investigated many potential clinical risk factors for GDM and compared the insulin sensitivity and beta-cell function across different gestational glucose tolerance groups. We also extensively evaluated the tolerance of the tests and preference of participants. However, some bias in recruitment is likely since <10% of the total pregnant population was recruited. Compared to the background Flemish pregnant population, participants were less often from an EM background, but rates of overweight and obesity were slightly higher [20].

A risk of a two-step approach is the delay or lack of follow-up of an abnormal GCT [22]. However, a Canadian population-based study showed that a universal two-step screening approach with a 50 g GCT was widely implemented, and that the OGTT was timely completed in 75% of women [10]. In our study, screening for GDM was performed between 24–28 weeks, in line with the general recommendations to have a high detection rate and to allow for sufficient time for the treatment of GDM to have an impact on pregnancy outcomes [23]. 

By accepting a low threshold for ruling out GDM, and a high threshold for diagnosing GDM on a screening test, the time and cost of a two-step approach for diagnosis could be reduced. The Canadian Diabetes Association recommends to diagnose GDM if the glucose level 1 h after the GCT is ≥ 11.1 mmol/L [24]. In our study, a GCT threshold of 10.2 mmol was the lowest cut-off, with a specificity of at least 99%. This threshold could potentially be used for a one-step diagnosis with the GCT, without the need of a subsequent OGTT in our population. However, several studies have shown a consistent lower positive predictive value of the GCT when performed in the afternoon [25,26,27]. The normal circadian decline in insulin sensitivity and/or beta-cell responsivity later in the day, may contribute to a positive GCT that might not have been positive if performed in the morning [25]. In contrast, in our study, the global interaction with the time of testing during the day of the GCT was not significant. 

Evaluation of the characteristics of the different groups according to the GCT and OGTT results, showed that the abnormal GCT GDM group with a GCT threshold ≥ 7.2 mmol/L had the highest risk profile, with more risk factors for GDM than the normal GCT GDM group. We also demonstrate that gestational glucose tolerance worsens from normal (normal GCT NGT) to mildly abnormal (abnormal GCT NGT) to GDM. The future risk to develop T2DM might gradually increase across these different groups, as has previously been shown in women with GDM based on the NDDG criteria [28]. The GCT might therefore help to identify women at a higher risk of developing glucose intolerance over time.

When using a universal one-step diagnostic approach with the 75 g OGTT, performing a FPG as an initial step and reserving a full OGTT for those with non-diagnostic FPG, or limiting the OGTT to 1 h cannot be recommended for our population, since the 2 h threshold contributed the most to the diagnosis of GDM. This might be different to other populations, where 50–75% of the diagnosis could be based on the FPG alone [29]. The reasons for the differences between populations may relate to the difference in the frequency of obesity and the degree of abnormal glucose metabolism in the background populations.

To establish the best strategy for diagnosing GDM to improve pregnancy outcomes, ideally, a large RCT should be performed, comparing different screening strategies, but this would be very challenging because of the very large sample size needed [30]. More research is also needed to evaluate the cost-effectiveness of different screening strategies based on the 2013 WHO criteria for GDM. In addition, more long-term data are needed on the risk to develop T2DM in women with GDM, based on the 2013 WHO criteria. Our cohort consisted mostly of a Caucasian population with a rather low background risk for GDM. The proposed modified two-step screening strategy might therefore only be feasible in lower-risk populations. 

## Figures and Tables

**Table 1:** The baseline characteristics and comparison of characteristics between the GDM and NGT group.

	Baseline Characteristics	GDM *N* = 231	NGT *N* = 1610	*p*-Value
**Mean gestational week first visit**	11.9 ± 1.8	11.9 ± 1.7	11.9 ±1.8	0.970
**Mean age (years)**	30.8 ± 4.1	32.0 ± 4.7	30.6 ± 3.9	**<0.001**
**Mean pre-pregnancy BMI (kg/m²)** **Pre-pregnancy overweight** **Pre-pregnancy obesity**	24.1 ± 4.7 22.2 (423) 11.3 (215)	25.8 ± 5.5 24.9 (54) 21.7 (47)	23.8 ± 4.4 21.8 (336) 9.1 (140)	**<0.001**
**Mean BMI at first visit (kg/m²)** **Overweight at first visit** **Obesity at first visit**	24.7 ± 4.7 25.2 (502) 13.3 (265)	26.6 ± 5.3 29.1 (67) 23.5 (54)	24.4 ± 4.5 24.9 (398) 11.0 (176)	**<0.001**
**Waist circumference at first visit (cm)** **Waist ≥ 80–88 cm** **Waist >88 cm**	87.3 ± 11.7 35.0 (671) 39.8 (762)	91.2 ± 13.0 30.7 (67) 50.9 (111)	86.5 ± 10.9 36.8 (567) 37.3 (574)	**<0.001**
**Ethnic minorities**	10.7 (213)	18.9 (43)	8.2 (132)	**<0.001**
**Highest education:** **Primary school** **Till 15 years** **High school** **Bachelor** **Master**	1.2 (24) 4.6 (92) 13.9 (278) 41.8 (806) 35.5 (684)	2.6 (6) 4.8 (11) 17.0 (36) 37.5 (81) 35.6 (77)	0.9 (15) 4.3 (69) 12.2 (189) 43.1 (675) 36.2 (568)	0.387
**Smoking before pregnancy**	29.5 (587)	35.1 (80)	28.4 (456)	**0.043**
**Smoking during pregnancy**	3.8 (75)	5.7 (13)	3.2 (52)	0.082
**First degree family history of diabetes**	13.1 (255)	18.7 (42)	11.8 (185)	**0.005**
**Second degree family history of diabetes**	44.1 (717)	48.3 (84)	42.6 (557)	0.166
History of GDM*	9.3 (90)	30.2 (36)	5.3 (40)	**<0.001**
**History of impaired glucose intolerance**	1.6 (27)	2.9 (6)	1.1 (15)	**0.033**
History of macrosomia*	11.7 (115)	15.8 (19)	11.5 (88)	0.176
**Systolic blood pressure first visit (mmHg)**	115.1 ± 10.7	116.4 ± 11.5	114.8 ± 10.4	**0.047**
**Diastolic blood pressure first visit (mmHg)**	70.6 ± 8.2	72.1 ± 8.8	70.3 ± 8.1	**0.002**
**Systolic hypertension first visit**	2.2 (44)	3.0 (7)	1.9 (30)	0.215
**Diastolic hypertension first visit**	1.9 (39)	3.5 (8)	1.6 (26)	0.063
**Systolic blood pressure at time of the OGTT (mmHg)**	113.4 ± 10.2	115.0 ± 11.3	113.1 ± 10.0	0.050
**Diastolic blood pressure at time of the OGTT (mmHg)**	67.3 ± 8.0	69.0 ± 8.3	67.0 ± 7.9	**<0.001**
**Systolic Hypertension at time of the OGTT**	1.2 (23)	3.1 (7)	0.9 (15)	**0.014**
**Diastolic Hypertension at time of the OGTT**	0.7 (13)	1.7 (4)	0.6 (9)	0.068
**Fertility treatment**	14.6 (292)	16.4 (38)	14.8 (238)	0.507
**PCOS**	7.1 (142)	4.8 (11)	7.3 (117)	0.169
**Multiparity**	47.7 (956)	51.9 (120)	46.5 (748)	0.120
**Fasting glycaemia (mmol/L) at first visit**	4.5 (4.2–4.7)	4.7 (4.4–4.9)	4.5 (4.4–4.7)	**<0.001**
**HbA1c (mmol/mol and %) at first visit**	31 (29–32) 5.0 (4.8–5.1)	32 (30–34) 5.1 (4.9–5.3)	30 (29–32) 4.9 (4.8–5.1)	**<0.001**
**Total cholesterol (mmol/L) at first visit**	4.7 (4.2–5.3)	6.3 (5.6–7.0)	6.3 (5.7–7.0)	0.894
**HDL-cholesterol (mmol/L) at first visit**	1.8 (1.5–2.0)	1.9 (1.6–2.2)	1.9 (1.6–2.2)	0.090
**LDL-cholesterol (mmol/L) at first visit**	2.5 (2.0–2.9)	3.4 (2.8–4.0)	3.4 (2.9–4.1)	0.611
**Triglycerides (mmol/L) at first visit**	1.1 (0.8–1.3)	2.0 (1.6–2.5)	1.8 (1.4–2.3)	**<0.001**
**HOMA-IR at first visit**	9.4 (6.6–13.5)	10.7 (7.9–16.9)	9.1 (6.5–12.9)	**<0.001**
**HOMA-B at first visit**	922.5 (669.9–1292.1)	930.0 (673.7–1334.0)	918.0 (667.8–1284.0)	0.492
**Mean gestational week GCT**	24.5 ± 0.9	24.6 ± 1.1	24.5 ± 0.9	0.439
**Timing of the GCT**	Before 12:00 a.m.: 44.4% (811) After 12:00 a.m.: 55.6% (1016)	Before 12:00 a.m.: 48.0% (109) After 12:00 a.m.: 52.0% (118)	Before 12:00 a.m.: 43.5% (684) After 12:00 a.m.: 56.5% (888)	0.201
**Hours after the last meal before the GCT**	3.6 ± 3.2	3.5 ± 3.1	3.6 ± 3.2	0.983
**Glucose value 1 h after the GCT (mmol/L)**	6.7 ± 1.5	8.1 ± 1.6	6.5 ± 1.4	**<0.001**
**Non-fasting glucose value before the GCT (mmol/L)**	4.9 ± 0.9	5.4 ± 1.1	4.9 ± 0.9	**<0.001**
**Mean gestational week OGTT**	26.9 ± 1.1	27.0 ± 1.2	26.9 ± 1.0	**0.037**
**Time between GCT and OGTT (weeks)**	2.4 ± 0.9	2.5 ± 1.1	2.3 ± 1.0	0.272
**OGTT (mmol/L):** **Fasting** **1 h** **2 h**	4.3 (4.1–4.6) 7.1 (6.0–8.3) 6.2 (5.3–7.2)	4.7 (4.4–5.1) 9.5 (8.5–10.3) 8.6 (7.5–9.1)	4.3 (4.1–4.5) 6.8 (5.9–7.8) 6.0 (5.1–6.9)	**<0.001** **<0.001** **<0.001**
**HbA1c (mmol/mol and %) at time of the OGTT**	30 (29–32) 4.9 (4.8–5.1)	32 (30–34) 5.1 (4.9–5.3)	30 (29–32) 4.9 (4.8–5.1)	**<0.001**
**Total cholesterol (mmol/L) at time of the OGTT**	6.3 (5.6–7.0)	6.3 (5.6-7.0)	6.3 (5.7–7.0)	0.894
**HDL-cholesterol (mmol/L) at time of the OGTT**	1.9 (1.6–2.2)	1.9 (1.6–2.2)	1.9 (1.6–2.2)	0.090
**LDL-cholesterol (mmol/L) at time of the OGTT**	3.4 (2.9–4.1)	3.4 (2.8–4.0)	3.4 (2.9–4.1)	0.611
**Triglycerides (mmol/L) at time of the OGTT**	1.8 (1.5–2.3)	2.0 (1.6–2.5)	1.8 (1.4–2.3)	**<0.001**
**HOMA-IR at time of the OGTT**	12.4 (8.9–17.7)	17.2 (11.5–26.5)	11.9 (8.6–16.7)	**<0.001**
**Matsuda index at time of the OGTT**	0.5 (0.4–0.8)	0.4 (0.3-0.5)	0.6 (0.4-0.8)	**<0.001**
**HOMA-B at time of the OGTT**	1561.8 (1122.9–2254.9)	1339.4 (1026.9–2073.9)	1584.0 (1132.0–2273.4)	0.087
**ISSI-2 at time of the OGTT**	0.14 (0.08–0.24)	0.09 (0.04–0.16)	0.14 (0.08–0.25)	**<0.001**
**Insulinogenic index/HOMA-IR at time of the OGTT**	0.31 (0.22–0.45)	0.21 (0.16–0.29)	0.33 (0.24–0.47)	**<0.001**

Baseline characteristics: characteristics of the whole cohort at 6–14 weeks of pregnancy and at the time of the GCT and OGTT; NGT: normal glucose tolerance; GDM: gestational diabetes mellitus; Categorical variables are presented as frequencies %(*n*); continuous variables are presented as mean ± SD if normally distributed and as median ± IQR if not normally distributed; overweight: body mass index (BMI) ≥ 25–29.9 kg/m²; obesity: BMI ≥ 30 kg/m²; hypertension: blood pressure systolic ≥ 140 mmHg or diastolic ≥ 90 mmHg; PCOS: polycystic ovarian syndrome; * A history of GDM and a history of a macrosomic baby (>4 kg) were calculated on the number of women with a previous pregnancy; At first visit: between 6–14 weeks of pregnancy; HDL: high-density lipoprotein cholesterol; LDL: low-density lipoprotein cholesterol; HOMA-IR: homeostatic model assessment of insulin resistance; HOMA-B: homeostatic model assessment of beta-cell function; ISSI-2: the insulin secretion-sensitivity index-2; *p*-value for comparisons between GDM and NGT; Differences are considered significant at *p*-value < 0.05.

**Table 2:** Comparison of the baseline characteristics of the ‘Belgian Diabetes in Pregnancy’ study (BEDIP) cohort with the pregnant Flemish background population.

	BEDIP-*N* Cohort *N* = 2006	Pregnant Background Population from 2014 *; *N* = 67,729
Mean age (years)	30.8 ± 4.1	28.7
≥40 years	2.0	2.7
Mean BMI at first visit (kg/m²)	24.7 ± 4.7	24.1 ± 4.6
Overweight	25.2	22.2
Obesity	13.3	11.2
Ethnic minorities **	10.7	15.2
Highest education **: Primary school Until 15 years High school Bachelor Master or PhD	1.2 4.6 13.9 41.8 35.5	4.1 7.3 34.9 27.3 18.9
Profession: ** Employee Laborer Self-employed No paid job (Unemployed, chronic ill or house wife)	63.6 5.1 7.1 9.4	58.8 14.0 5.5 18.3
Multiparity	47.7	55.8
Fertility treatment	14.6	6.9
Hypertension	4.1	4.6

Categorical variables are presented as frequencies %(*n*); continuous variables are presented as mean ± SD;* Data from the pregnant background population were retrieved from the Flemish ‘Study Centre for Perinatal Epidemiology’ (SPE) database from 2014 [20]; ** These variables were retrieved from the 2014 report of the Care and Health department, a Flemish governmental institution; overweight: body mass index (BMI) ≥ 25–29.9 kg/m²; obesity: BMI ≥ 30 kg/m²; hypertension: blood pressure systolic ≥ 140 mmHg, or diastolic ≥ 90 mmHg.

**Table 3:** Differences in characteristics across the four gestational tolerance groups according to the GCT and OGTT result.

	GDM with GCT ≥ 7.2 mmol/L Group 1 *N* = 165	GDM with GCT < 7.2 mmol/L Group 2 *N* = 63	NGT with GCT ≥ 7.2 mmol/L Group 3 *N* = 472	NGT with GCT < 7.2 mmol/L Group 4 *N* = 1113	*p*-Value 1 vs. 4	*p*-Value 2 vs. 4	*p*-Value 3 vs. 4
**Age (years)**	32.5 ± 4.7	31.1 ± 4.1	31.1 ± 3.9	30.4 ±3.9	**<0.001**	0.13	**0.004**
**≥ 40 years**	9.7 (16)	0% (0)	1.3 (6)	1.3 (15)	**<0.001**	1.00	1.00
**BMI (Kg/m²) at first visit**	26.5 ± 5.2	26.8 ± 5.7	24.5 ± 4.5	24.3 ± 4.4	**<0.001**	**<0.001**	0.65
**Overweight at first visit** **Obesity at first visit**	28.7 (47) 24.8 (39)	30.2 (19) 23.8 (15)	24.5 (115) 12.1 (57)	24.7 (273) 10.5 (116)	**<0.001**	**<0.001**	0.84
**Waist circumference at first visit (cm)**	90.9 ± 12.5	91.9 ± 14.3	86.4 ±11.5	86.6 ± 10.6	**<0.001**	**0.006**	0.39
**Waist ≥80–88 cm** **Waist > 88 cm**	28.8 (45) 52.6 (82)	33.9 (20) 47.5 (28)	38.2 (174) 34.4 (157)	36.4 (387) 38.7 (411)	**0.004**	0.35	0.28
**BMI (Kg/m²) at time of OGTT**	29.1 ± 5.0	29.3 ± 5.7	27.0 ± 4.4	26.9 ± 4.4	**<0.001**	**0.001**	0.88
**Overweight at time of OGTT** **Obesity at time of OGTT**	37.7 (60) 37.1 (59)	44.1 (26) 33.9 (20)	37.1 (173) 22.1 (103)	41.3 (444) 20.6 (222)	**<0.001**	**0.004**	0.50
**Ethnic minorities**	18.4 (30)	19.3 (12)	7.7 (36)	8.2 (91)	**0.003**	**<0.001**	0.80
**Highest education:** **primary school** **Until 15 years** **High school** **Bachelor** **Master**	2.5 (4) 3.7 (6) 25.9 (42) 37.5 (57) 34.9 (53)	1.6 (1) 6.4 (4) 14.5 (9) 39.3 (24) 39.3 (24)	0.4 (2) 4.3 (20) 17.2 (79) 41.4 (190) 38.6 (177)	1.2 (13) 4.1 (46) 16.1 (175) 44.0 (478) 35.2 (382)	0.41	0.78	0.51
**History of smoking before pregnancy**	33.7 (55)	38.7 (24)	28.1 (132)	28.7 (318)	0.19	0.09	0.82
**Smoking during pregnancy**	6.7 (11)	1.6 (1)	4.3 (20)	2.8 (31)	**0.02**	1.00	0.16
**First degree family history of diabetes**	20.8 (32)	12.7 (7)	12.9 (60)	11.3 (122)	**0.005**	0.09	0.37
**Second degree family history of diabetes**	50.4 (64)	44.4 (20)	45.3 (172)	41.7 (379)	0.06	0.72	0.24
**First degree family history of GDM**	7.3 (11)	5.4 (3)	5.0 (22)	3.7 (38)	**0.04**	0.50	0.24
**History of GDM**	36.0 (32)	13.8 (4)	7.3 (16)	4.6 (24)	**<0.001**	**0.03**	0.13
**History of impaired glucose intolerance**	3.4 (5)	1.8 (1)	1.7 (7)	0.9 (8)	**0.02**	0.40	0.26
**History of macrosomia**	20.0 (18)	3.4 (1)	10.9 (24)	11.9 (63)	**0.04**	0.23	0.71
**Systolic blood pressure first visit (mmHg)**	116.5 ± 11.7	116.0 ± 11.3	115.4 ± 10.9	114.6 ± 10.1	0.06	0.14	0.11
**Diastolic blood pressure first visit (mmHg)**	72.5 ± 9.0	71.4 ± 8.4	71.2 ± 8.6	70.0 ± 7.8	**<0.001**	0.09	**0.007**
**Systolic blood pressure at time of OGTT (mmHg)**	115.1 ± 11.1	114.6 ± 11.8	113.0 ± 10.2	113.1 ± 10.0	0.08	0.40	0.84
**Diastolic blood pressure at time of the OGTT (mmHg)**	68.7 ± 7.9	69.8 ± 9.1	67.0 ± 8.2	66.9± 7.8	**0.007**	**0.004**	0.85
**Fertility treatment**	20.0 (33)	7.9 (5)	16.7 (79)	13.8 (154)	**0.04**	0.18	0.14
**PCOS**	4.8 (8)	4.8 (3)	8.1 (38)	6.8 (76)	0.33	0.52	0.37
**Multiparity**	54.5 (90)	46.0 (29)	46.4 (219)	45.9 (510)	**0.04**	0.98	0.84
**Fasting glycaemia (mmol/L) at first visit**	4.7 (4.4–4.9)	4.6 (4.3–4.9)	4.5 (4.3–4.7)	4.5 (4.3–4.7)	**<0.001**	**0.004**	0.49
**HbA1c (mmol/mol and %) at first visit**	32 (30–33) 5.1 (4.9–5.2)	31 (30–34) 5.0 (4.9–5.3)	31 (28–32) 5.0 (4.8–5.1)	31 (28–32) 5.0 (4.8–5.1)	**<0.001**	**0.01**	0.44
**Total cholesterol (mmol/L) at first visit**	4.8 (4.2–5.4)	4.8 (4.3–5.4)	4.8 (4.2–5.4)	4.6 (4.1–5.2)	**0.01**	0.12	**0.008**
**HDL-cholesterol (mmol/L) at first visit**	1.7 (1.5–2.0)	1.8 (1.5–2.0)	1.8 (1.5–2.0)	1.7 (1.5–2.0)	0.59	0.68	0.08
**LDL-cholesterol (mmol/L) at first visit**	2.4 (2.1–2.9)	2.4 (2.1–2.9)	2.4 (2.0–2.9)	2.4 (2.0–2.8)	0.10	0.20	0.43
**Triglycerides (mmol/L) at first visit**	1.2 (0.9–1.5)	1.0 (0.8–1.3)	1.0 (0.8–1.3)	1.0 (0.8–1.2)	**<0.001**	**0.04**	**0.02**
**HOMA-IR at first visit**	10.5 (7.8–17.1)	12.0 (8.3–14.6)	9.2 (6.2–14.1)	9.07 (6.5–12.7)	**<0.001**	**<0.001**	0.33
**HOMA-B at first visit**	1354.1 (641.7–1328.4)	969.4 (776.8–1339.6)	936.0 (662.7–1368.0)	912.0 (668.8–1273.3)	0.92	0.17	0.44
**OGTT (mmol/L)** **Fasting** **1 h** **2 h**	4.7 (4.4–5.1) 9.7 (8.7–10.5) 8.8 (7.9–9.2)	4.7 (4.3–5.2) 8.8 (7.8–9.9) 8.5 (6.7–9.0)	4.3 (4.1–4.5) 7.5 (6.5–8.4) 6.5 (5.5–7.4)	4.3 (4.1–4.5) 6.5 (5.7–7.5) 5.8 (5.0–6.6)	**<0.001** **<0.001** **<0.001**	**<0.001** **<0.001** **<0.001**	0.18 **<0.001** **<0.001**
**HbA1c ( mmol/mol and %) at time of the OGTT**	32 (30–34) 5.1 (4.9–5.3)	31 (28–33) 5.0 (4.8–5.2)	30 (28–32) 4.9(4.8–5.1)	30 (28–32) 4.9 (4.8–5.1)	**<0.001**	**<0.001**	0.13
**Total cholesterol (mmol/L) at time of the OGTT**	6.3 (5.6–7.0)	6.3 (5.6–7.1)	6.1 (5.6–7.0)	6.3 (5.7–7.1)	0.57	0.99	0.02
**HDL-cholesterol (mmol/L) at time of the OGTT**	1.9 (1.6–2.2)	1.9 (1.6–2.2)	1.9 (1.6–2.2)	1.9 (1.6–2.2)	0.08	0.44	0.44
**LDL-cholesterol (mmol/L) at time of the OGTT**	3.4 (2.8–4.0)	3.5 (3.0–4.3)	3.3 (2.8–4.0)	3.5 (2.9–4.2)	0.14	0.99	**0.004**
**Triglycerides (mmol/L) at time of the OGTT**	2.1 (1.6–2.7)	2.0 (1.6–2.3)	1.8 (1.4–2.3)	1.8 (1.4–2.2)	**<0.001**	**0.04**	0.22
**HOMA-IR at time of the OGTT**	17.1 (11.8–26.1)	17.4 (11.1–28.5)	12.0 (8.6–17.5)	11.9 (8.6–16.4)	**<0.001**	**<0.001**	**0.004**
**Matsuda index at time of the OGTT**	0.38 (0.26–0.5)	0.39 (0.24–0.53)	0.54 (0.39–0.75)	0.61 (0.44–0.82)	**<0.001**	**<0.001**	**<0.001**
**HOMA-B at time of the OGTT**	1315.8 (1001.2–2069.5)	1501.2 (1048.5–2268.0)	1592.1 (1155.3–2309.7)	1584.0 (1125.0–2259.0)	0.29	0.36	0.13
**ISSI-2 at time of the OGTT**	0.10 (0.05–0.16)	0.09 (0.04–0.17)	0.13 (0.07–0.23)	0.15 (0.09–0.26)	**<0.001**	**0.009**	**0.04**
**Insulinogenic index/HOMA-IR at time of the OGTT**	0.21 (0.15–0.29)	0.21 (0.17–0.30)	0.31 (0.22–0.46)	0.34 (0.24–0.48)	**<0.001**	**<0.001**	**0.004**

GCT: glucose challenge test; NGT: normal glucose tolerance; GDM: gestational diabetes mellitus; Categorical variables are presented as frequencies %(*n*); continuous variables are presented as mean ± SD if normally distributed and as median ± IQR if not normally distributed; overweight: body mass index: BMI ≥ 25–29.9 kg/m²; obesity: BMI ≥ 30 kg/m²; PCOS: polycystic ovarian syndrome; HDL: high-density lipoprotein cholesterol; LDL: low-density lipoprotein cholesterol; HOMA-IR: homeostatic model assessment of insulin resistance; HOMA-B: homeostatic model assessment of beta-cell function; ISSI-2: the insulin secretion- sensitivity index-2; A history of GDM and a history of a macrosomic baby (>4 kg) were calculated on the number of women with a previous pregnancy; Differences are considered significant at *p*-value < 0.05.

**Table 4:** Sensitivity and specificity of the GCT using a threshold of 7.2 mmol/L combined with clinical risk factors.

Risk factors	Total Number of OGTT’s needed with GCT and Risk Factors combined % (*n*)	Number of OGTT’s needed based on GCT % (*n*)	Number of OGTT’s needed based on Risk Factors % (*n*)	% (*n*) GDM	Sensitivity (95% CI), % *n*/*N*	Specificity (95% CI), % *n*/N	LR+ (95% CI)	LR− (95% CI)	Positive Posttest Probability	Negative Posttest Probability
**EM background**	41.3 (749)	31.2 (566)	10.0 (182)	9.8 (178)	78.1 (72.1–83.3) 178/228	64.0 (61.6–66.3) 1282/1583	2.2 (2.0–2.4)	0.34 (0.27–0.44)	24.3% (20.0–28.7)	4.8% (4.0–5.9)
**BMI ≥ 30 kg/m²**	40.9 (741)	30.7 (557)	10.1 (184)	9.7 (176)	77.2 (71.2–82.5) 176/228	64.3 (61.9–66.7) 1020/1583	2.2 (2.0–2.4)	0.35 (0.28–0.45)	24.3% (20.0–28.7)	5.0% (4.1–6.0)
**History of GDM**	36.7 (665)	32.5 (589)	4.2 (76)	9.3 (169)	74.1 (67.9–79.7) 169/228	68.7 (66.4–71.0) 1089/1583	2.4 (2.1–2.6)	0.38 (0.30–0.47)	26.0% (21.4–30.7)	5.3% (4.4–6.4)
**Any of the 3 risk factors**	47.6 (868)	25.5 (462)	22.1 (400)	10.4 (189)	82.9 (77.4–87.5) 189/228	57.5 (55.0–59.9) 911/1583	1.9 (1.8–2.1)	0.30 (0.22–0.40)	22.4% (18.4–26.5)	4.2% (3.4–5.2)

GCT: glucose challenge test; OGTT: oral glucose tolerance test; GDM: gestational diabetes mellitus; EM: ethnic minority; BMI: Body mass index in kg/m²; CI: confidence interval; Sensitivity: *n* = number with GCT ≥ cut-off and *N* = number with GDM; Specificity: *n* = number with GCT < cut-off, and *N* = number without GDM; LR+: positive likelihood ratio; LR−: negative likelihood ratio.
